# Midregional pro-Adrenomedullin in addition to b-type natriuretic peptides in the risk stratification of patients with acute dyspnea: an observational study

**DOI:** 10.1186/cc7975

**Published:** 2009-07-23

**Authors:** Mihael Potocki, Tobias Breidthardt, Tobias Reichlin, Nils G Morgenthaler, Andreas Bergmann, Markus Noveanu, Nora Schaub, Heiko Uthoff, Heike Freidank, Lorenz Buser, Roland Bingisser, Michael Christ, Alexandre Mebazaa, Christian Mueller

**Affiliations:** 1 Department of Internal Medicine, University Hospital, Petersgraben 4, 4031 Basel, Switzerland; 2 Research Department, B.R.A.H.M.S. AG, Neuendorfstrasse 25, 16761 Hennigsdorf/Berlin, Germany; 3 Department of Laboratory Medicine, University Hospital, Petersgraben 4, 4031 Basel, Switzerland; 4 Internal Medicine, Klinikum Nuernberg, Prof.-Ernst-Nathan-Str. 1, 90419 Nuernberg, Germany; 5 APHP, Hôpital Lariboisière University Paris 7 Diderot, 75010 Paris, France

## Abstract

**Introduction:**

The identification of patients at highest risk for adverse outcome who are presenting with acute dyspnea to the emergency department remains a challenge. This study investigates the prognostic value of the newly described midregional fragment of the pro-Adrenomedullin molecule (MR-proADM) alone and combined to B-type natriuretic peptide (BNP) or N-terminal proBNP (NT-proBNP) in patients with acute dyspnea.

**Methods:**

We conducted a prospective, observational cohort study in the emergency department of a University Hospital and enrolled 287 unselected, consecutive patients (48% women, median age 77 (range 68 to 83) years) with acute dyspnea.

**Results:**

MR-proADM levels were elevated in non-survivors (n = 77) compared to survivors (median 1.9 (1.2 to 3.2) nmol/L vs. 1.1 (0.8 to 1.6) nmol/L; *P *< 0.001). The areas under the receiver operating characteristic curve (AUC) to predict 30-day mortality were 0.81 (95% CI 0.73 to 0.90), 0.76 (95% CI 0.67 to 0.84) and 0.63 (95% CI 0.53 to 0.74) for MR-proADM, NT-proBNP and BNP, respectively (MRproADM vs. NTproBNP *P *= 0.38; MRproADM vs. BNP *P *= 0.009). For one-year mortality the AUC were 0.75 (95% CI 0.69 to 0.81), 0.75 (95% CI 0.68 to 0.81), 0.69 (95% CI 0.62 to 0.76) for MR-proADM, NT-proBNP and BNP, respectively without any significant difference. Using multivariate linear regression analysis, MR-proADM strongly predicted one-year all-cause mortality independently of NT-proBNP and BNP levels (OR = 10.46 (1.36 to 80.50), *P *= 0.02 and OR = 24.86 (3.87 to 159.80) *P *= 0.001, respectively). Using quartile approaches, Kaplan-Meier curve analyses demonstrated a stepwise increase in one-year all-cause mortality with increasing plasma levels (*P *< 0.0001). Combined levels of MR-proADM and NT-proBNP did risk stratify acute dyspneic patients into a low (90% one-year survival rate), intermediate (72 to 82% one-year survival rate) or high risk group (52% one-year survival rate).

**Conclusions:**

MR-proADM alone or combined to NT-proBNP has a potential to assist clinicians in risk stratifying patients presenting with acute dyspnea regardless of the underlying disease.

## Introduction

Acute dyspnea is a frequent clinical presentation in the emergency department (ED). Cardiac and pulmonary disorders account for more than 75% of patients presenting with acute dyspnea to the ED [1,2]. The identification of patients at highest risk for adverse outcomes with acute dyspnea remains a challenge. Patient history and physical examination remain the cornerstone of clinical evaluation [3], while disease-specific scoring tools [4,5] and biomarkers such as natriuretic peptides have been introduced to assist the clinician in the diagnostic and prognostic assessment [6-9].

Adrenomedullin (ADM) is a peptide of 52 amino acids and was originally isolated from human pheochromocytoma cells and has later been detected in other tissues, including heart, adrenal medulla, lungs, and kidneys [10,11]. It is a potent vasodilator, causes hypotension and has inotropic and natriuretic effects stimulated by cardiac pressure and volume overload [12,13]. The midregional fragment of the pro-Adrenomedullin molecule (MR-proADM), consisting of amino acids 24 to 71, is more stable than ADM itself, is secreted in equimolar amounts to ADM, and is easier to measure [14]. Elevated levels of ADM have frequently been reported in patients with various diseases. In patients with sepsis, pneumonia, chronic obstructive pulmonary disease, myocardial infarction, and heart failure, MR-proADM levels were elevated and predicted mortality [15-20]. In order to be relevant, a marker should provide prognostic information reflective of the wide spectrum of diseases that might be present among patients with acute dyspnea. In clinical practice, the identification of dyspneic patients at highest risk for adverse outcomes is an unmet clinical need. Accordingly, in an effort to better understand the role of MR-proADM in this setting, we tested the individual and combined prognostic utility of MR-proADM together with established prognostic predictors such as B-type natriuretic peptide (BNP) or N-terminal proBNP (NT-proBNP).

## Materials and methods

### Study population

From April 2006 to March 2007, we prospectively enrolled 287 unselected, consecutive patients with acute dyspnea as the most prominent symptom presenting to the ED of the University Hospital Basel, Switzerland. Patients under 18 years of age, patients on hemodialysis and trauma patients were excluded. The study was carried out according to the principles of the Declaration of Helsinki and approved by the local ethics committee. Written informed consent was obtained from all participating patients.

### Clinical evaluation and follow-up

Patients underwent an initial clinical assessment including clinical history, physical examination, echocardiogram, pulse oximetry, blood tests including BNP, and chest X-ray. Echocardiography and pulmonary function tests were performed according to the treating physician.

Two independent internists reviewed all medical records including BNP levels and independently classified the patient's primary diagnosis into seven categories: acute decompensated heart failure (ADHF), acute exacerbation of chronic obstructive pulmonary disease (AECOPD), pneumonia, acute complications of malignancy, acute pulmonary embolism, hyperventilation, and others. In the event of diagnostic disagreement among the internist reviewers, they were asked to meet to come to a common conclusion. In the event that they were unable to come to a common conclusion, a third-party internist adjudicator was asked to review the data and determine which diagnosis was the most accurate.

The endpoint of the present study was defined as one-year all-cause mortality. Each patient was contacted for follow-up, via telephone, by a single trained researcher at specified intervals. Regarding mortality data, referring physicians were contacted or the administrative databases of respective hometowns were reviewed, if necessary. Of note, one patient was lost to follow-up, so mortality analyses were performed in 286 patients.

### Laboratory measurements

Blood samples for determination of MR-proADM, BNP, and NT-proBNP were collected at presentation into tubes containing potassium EDTA. Samples were frozen at -80°C until later analysis. MR-proADM was detected with a sandwich immunoluminometric assay (MR-proADM, BRAHMS AG, Hennigsdorf/Berlin, Germany), as described elsewhere [14]. Mean MR-proADM in 264 healthy individuals in previous investigations was 0.33 ± 0.07 nmol/L (range, 0.10–0.64 nmol/L) and the assay has a measuring range from 0 to 100 nmol/L. The limit of detection and limit of quantification were 0.05 and 0.23 nmol/L, respectively. The intra assay CV was 1.9% and the inter laboratory CV was 9.8%. NT-proBNP levels were determined by a quantitative electrochemiluminescence immunoassay (Elecsys proBNP, Roche Diagnostics AG, Zug, Switzerland) [21]. BNP was measured by a microparticle enzyme immunoassay (AxSym, Abbott Laboratories, Abbott Park/IL, USA) [22].

### Statistical analysis

Continuous variables are presented as mean ± standard deviation or median (with interquartile range), and categorical variables as numbers and percentages. Univariate data on demographic and clinical features were compared by Mann-Whitney U test or Fisher's exact test as appropriate. Correlations among continuous variables were assessed by the Spearman rank-correlation coefficient. Plasma levels of MR-proADM, NT-proBNP, and BNP were log-transformed to achieve a normal distribution. Receivers operating characteristic (ROC) curves were utilized to evaluate the accuracy of MR-proADM, NT-proBNP, and BNP to predict death at one year and areas under the curve (AUC) were calculated for all markers. AUCs were compared according to the method by Hanley and McNeil [23]. To identify independent predictors of outcome, linear regression analysis was assessed by univariate and multivariate analysis. Factors with univariate significance of *P *< 0.1 were included in multivariate analysis. To test whether higher MR-proADM levels in non-survivors are found regardless of the underlying diagnosis, linear regression analysis for one-year mortality was performed to exclude the possibility of confounding. We considered the variables MR-proADM and diagnosis in parallel in the same linear regression model to look for interaction terms. The Kaplan-Meier cumulative survival curves in which patients were divided into quartiles of biomarker plasma levels were constructed and compared by the log-rank test. Glomerular filtration rate was calculated using the abbreviated Modification of Diet in Renal Disease formula [24]. Data were statistically analyzed with SPSS 15.0 software (SPSS Inc, Chicago, IL, USA) and the MedCalc 9.3.9.0 package (MedCalc Software, Mariakerke, Belgium). All probabilities were two tailed and *P *< 0.05 was regarded as significant.

## Results

### Patient characteristics

The demographic features of the 287 acute dyspneic patients, at admission in the ED, are shown in Table 1. The primary diagnosis was ADHF in 154 (54%) patients, AECOPD in 57 (20%) patients, pneumonia in 32 (11%) patients, acute pulmonary embolism in 8 (3%) patients, acute complications of malignancy in 7 (2%) patients, hyperventilation in 5 (2%) patients, and other causes such as interstitial lung disease, asthma, or bronchitis in 24 (8%) patients. Diuretics (52%) were the most common oral chronic medications recorded at admission, followed by angiotensin converting enzyme inhibitors or angiotensin-receptor blockers (49%), and beta-blockers (39%).

**Table 1:** Patients' characteristics

Characteristic	All patients (n = 287)
Age, years^*a*^	77 (68 to 83)
Male gender (%)	52
Body mass index – kg/m^2 *b*^	26 ± 6
*History (%)*	
Hypertension	68
History of heart failure	24
Coronary artery disease	28
Diabetes mellitus	18
Chronic obstructive pulmonary disease	34
Chronic kidney disease	28
*Shortness of breath (%)*	
While walking up a slight incline (NYHA II)	20
While walking on level ground (NYHA III)	40
At rest (NYHA IV)	40
*Physical examination findings (%)*	
Heart rate, bpm^*b*^	93 ± 23
Systolic blood pressure, mmHg^*b*^	138 ± 26
Diastolic blood pressure, mmHg^*b*^	83 ± 16
Respiratory rate^*a*^	24 (20 to 28)
Rales	54
Lower extremity edema	42
Hepatojugular reflux	8
Jugular venous distension	28
*Oral chronic medication on admission (%)*	
Beta-blockers	39
Angiotensin-converting enzyme inhibitor/Angiotensin-II-receptor blockers	49
Loop diuretics	52
Calcium antagonists	18
Digoxin	5
Spironolactone	2
*Laboratory findings*	
Serum creatinine, μmol/L^*a*^	85 (66 to 120)
eGFR, mL/min/1.73 m^*2*^^*a*^	67 (44 to 89)
Blood urea nitrogen, mmol/L^*a*^	7.3 (5.4 to 12.0)
Sodium, mmol/L^*a*^	137 (134 to 139)
Hemoglobin, g/L^*a*^	133 (118 to 145)
Troponin T, μg/L^*a *^(n = 192)	0.01 (0.01 to 0.03)
BNP, pg/mL^*a*^	349 (90 to 1120)
NT-proBNP, pg/mL^*a*^	1656 (314 to 6105)
MR-proADM, nmol/L^*a*^	1.2 (0.8 to 2.0)
*Echocardiography findings (%)*	*(n = 116)*
Left ventricular ejection fraction^*a*^	56 (35 to 65)

^*a *^median (interquartile range), ^*b *^means ± standard deviation.

BNP = B-type natriuretic peptide; eGRF = estimated glomerular filtration rate; MR-proADM = midregional pro-adrenomedullin; NT-proBNP = N-terminal pro-B-type natriuretic peptide; NYHA = New York Heart Association.

### MR-proADM levels at admission

**Figure 1:**
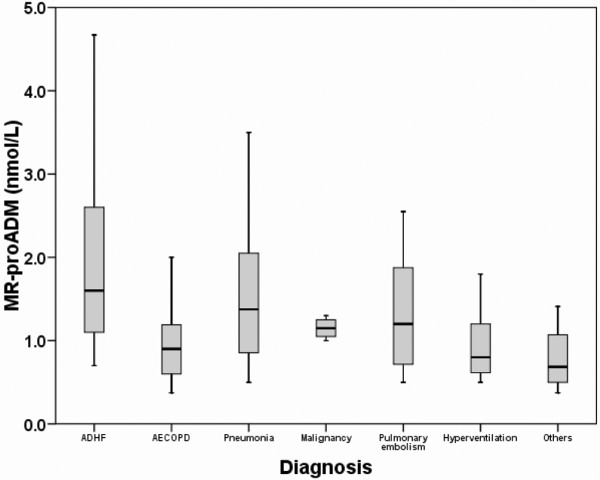
Midregional pro-adrenomedullin concentrations at admission as a function of diagnosis. ADHF = acute decompensated heart failure; AECOPD = acute exacerbation of chronic obstructive pulmonary disease; MR-proADM = midregional pro-adrenomedullin.

The median plasma level of MR-proADM on admission was 1.2 nmol/L (0.8 to 2.0 nmol/L) in all patients. Levels were higher in patients admitted with ADHF (1.6 (1.1 to 2.6) nmol/L) than in patients with AECOPD (0.8 (0.6 to 1.1) nmol/L; *P *< 0.001) and pneumonia (1.2 (0.9 to 2.0) nmol/L; *P *= 0.015, Figure 1).

In addition, plasma levels of MR-proADM were higher in patients with a history of hypertension (*P *< 0.001), renal insufficiency (*P *< 0.001), coronary artery disease (*P *= 0.004), and diabetes mellitus (*P *= 0.038) but similar between women and men. Plasma MR-proADM on admission correlated with age (r_s _= 0.51, *P *< 0.001), estimated glomerular filtration rate (r_s _= -0.76, *P *< 0.001), BNP (r_s _= 0.63, *P *< 0.01), and NTproBNP (r_s _= 0.75, *P *< 0.001), whereas only a weak correlation was found with New York Heart Association class (r_s _= 0.29, *P *< 0.001).

### Prediction of death by MR-proADM and natriuretic peptides

**Figure 2:**
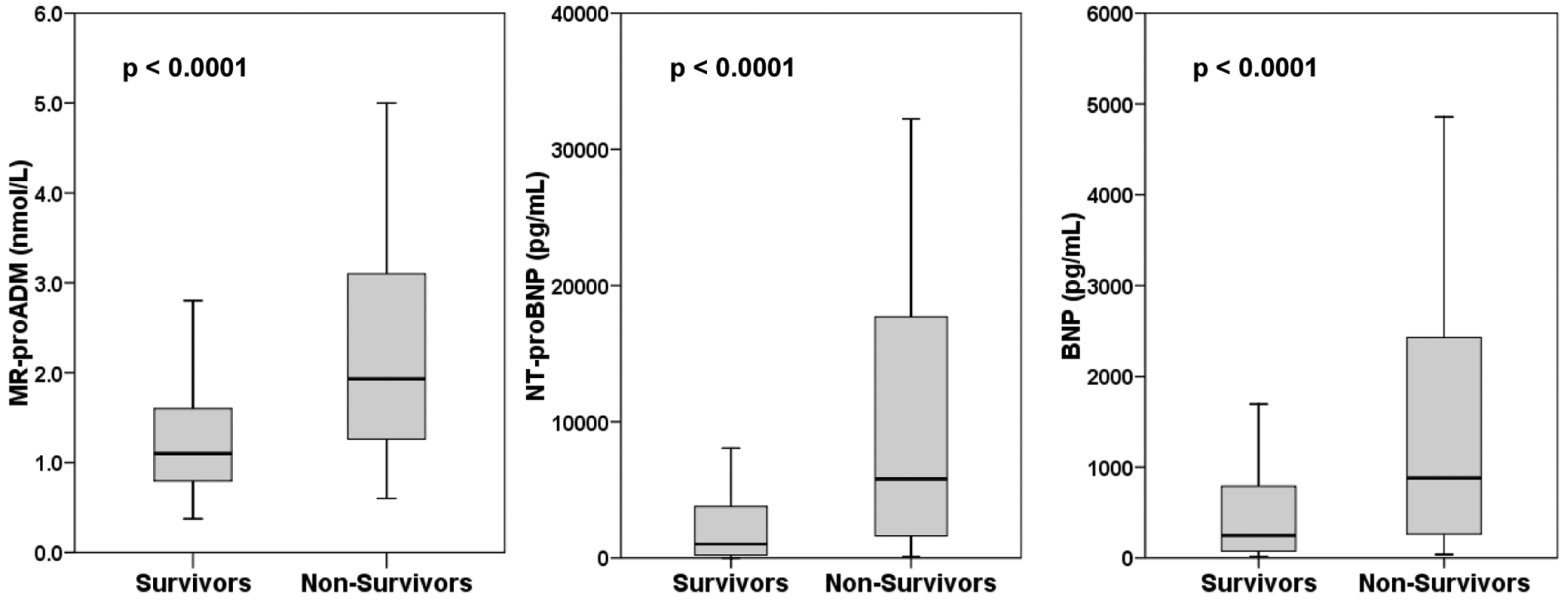
Midregional pro-adrenomedullin, N-terminal pro B-type natriuretic peptide and B-type natriuretic peptide concentrations at admission as a function of survival at one year. BNP = B-type natriuretic peptide; MR-proADM = midregional pro-adrenomedullin; NT-proBNP = N-terminal pro-B-type natriuretic peptide.

Seventy-seven patients (27%) reached the endpoint of one-year all-cause mortality. Figure 2 illustrates that non-survivors had higher MR-proADM levels with a median of 1.9 (1.2 to 3.2) nmol/L than survivors with a median of 1.1 (0.8 to 1.6) nmol/L (*P *< 0.001). The BNP and NT-proBNP levels were also higher in non-survivors than in survivors (881 (258 to 2436) pg/mL and 5803 (1608 to 17,908) pg/mL vs. 248 (73 to 803) pg/mL and 1015 (213 to 3904) pg/mL; *P *< 0.001 for both). The pattern of higher MR-proADM concentrations in non-survivors versus survivors remained when analysis were repeated in patients without (1.23 vs. 0.85 nmol/L; *P *= 0.001) or with (2.30 vs. 1.30 nmol/L; *P *< 0.001) ADHF. To test whether higher MR-proADM levels are found in non-survivors regardless of the underlying diagnosis, linear regression analysis for one-year mortality was performed and showed no significant interaction between MR-proADM levels and diagnosis.

**Figure 3:**
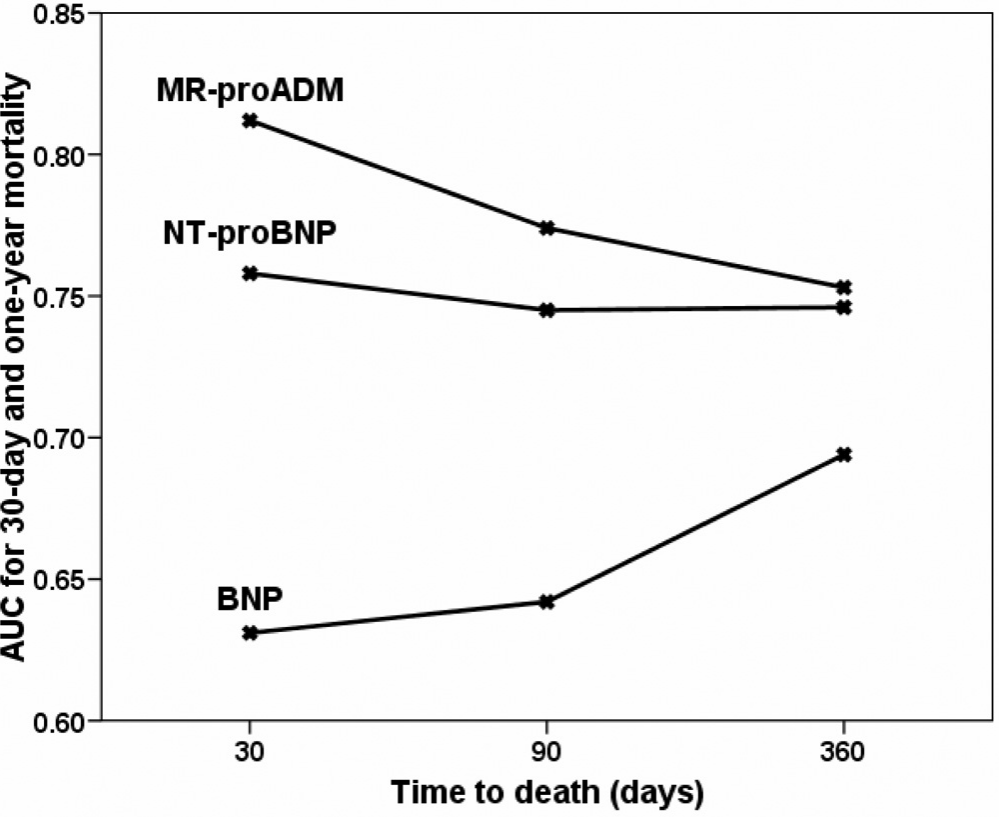
Area under the receiver-operating characteristic curve for midregional pro-adrenomedullin, N-terminal pro B-type natriuretic peptide and B-type natriuretic peptide to predict 30-day and one-year mortality. AUC = area under the receiver-operating characteristic curve; BNP = B-type natriuretic peptide; MR-proADM = midregional pro-adrenomedullin; NT-proBNP = N-terminal pro-B-type natriuretic peptide.

The results of the ROC analysis are showed in Figure 3. The AUC to predict 30- and 90-day mortality were 0.81 (95% confidence interval (CI) 0.73 to 0.90) and 0.77 (95% CI 0.70 to 0.85) for MR-proADM, 0.76 (95% CI 0.67 to 0.84) and 0.75 (95% CI 0.67 to 0.82) for NT-proBNP and 0.63 (95% CI 0.53 to 0.74), and 0.64 (95% CI 0.56 to 0.73) for BNP. There was a significant difference between the AUC for MR-proADM and the AUC for BNP for 30-day (*P *= 0.009) and 90-day (*P *= 0.02) mortality. The ROC analysis for one-year mortality demonstrated an AUC for MR-proADM of 0.75 (95% CI 0.69 to 0.81), for NT-proBNP of 0.75 (95% CI 0.68 to 0.81) and for BNP of 0.69 (95% CI 0.62 to 0.76). There was no significant difference between the AUC of MR-proADM and NT-proBNP (*P *= 0.91) or between MR-proADM and BNP (*P *= 0.21).

### Incremental value of MR-proADM

Linear regression analysis showed that plasma levels of MR-proADM, NT-proBNP, BNP, diagnosis of ADHF, New York Heart Association class, age, and estimated glomerular filtration rate, all assessed on ED admission, were predictors of one-year all-cause mortality (Table 2). The multivariate analysis was conducted with two separate models, one including NT-proBNP and the other including BNP. In the model with NT-proBNP, only MR-proADM, NT-proBNP, and age remained significant predictors with the highest odds ratio (OR) for MR-proADM (OR = 10.46 (1.36 to 80.50), *P *= 0.02). In the model with BNP, only MR-proADM (OR = 24.86 (3.87 to 159.80), *P *= 0.001) and age independently predicted one-year all-cause mortality in our acutely dyspneic patients (Table 3).

**Table 2:** Logistic regression analysis for one-year all-cause mortality

Variable	Odds ratio (95% CI)	*P *value
**MR-proADM^*a*^**	38.64 (12.13 to 123.14)	<0.001
**NT-proBNP^*a*^**	3.46 (2.29 to 5.28)	<0.001
**BNP^*a*^**	3.04 (1.95 to 4.75)	<0.001
**Age**	1.07 (1.04 to 1.11)	<0.001
**eGRF^*a*^**	0.12 (0.04 to 0.33)	<0.001
**Diagnosis of ADHF**	2.58 (1.48 to 4.51)	0.001
**NYHA class**	1.77 (1.23 to 2.57)	0.002
**Arterial hypertension**	1.28 (0.72 to 2.26)	0.40
**Male gender**	1.24 (0.73 to 2.10)	0.42
**History of coronary artery disease**	0.88 (0.49 to 1.58)	0.66
**Diabetes mellitus**	0.89 (0.45 to 1.78)	0.74

^*a *^log-transformed to achieve normal distribution.

ADHF = acute decompensated heart failure; BNP = B-type natriuretic peptide; CI = confidence interval; eGRF = estimated glomerular filtration rate; MR-proADM = midregional pro-adrenomedullin; NT-proBNP = N-terminal pro-B-type natriuretic peptide; NYHA = New York Heart Association.

**Table 3:** Multivariable logistic regression analysis for one-year all-cause mortality

	Model with NTproBNP		Model with BNP	
		
Variable	Odds ratio (95% CI)	*P *value	Odds ratio (95% CI)	*P *value
**MR-proADM^*a*^**	10.46 (1.36 to 80.50)	0.02	24.86 (3.87 to 159.80)	0.001
**NT-proBNP^*a*^**	2.90 (0.35 to 6.25)	0.006	-	-
**BNP^*a*^**	-	-	1.90 (0.89 to 4.10)	0.10
**Diagnosis of ADHF**	0.44 (0.18 to 1.07)	0.07	0.59 (0.23 to 1.47)	0.26
**NYHA class**	1.48 (0.95 to 2.30)	0.08	1.35 (0.88 to 2.08)	0.17
**Age**	1.04 (1.01 to 1.08)	0.02	1.04 (1.01 to 1.08)	0.01
**eGRF^*a*^**	4.48 (0.90 to 22.30)	0.07	3.40 (0.72 to 16.04)	0.12

^*a *^log-transformed to achieve normal distribution.

ADHF = acute decompensated heart failure; BNP = B-type natriuretic peptide; CI = confidence interval; eGRF = estimated glomerular filtration rate; MR-proADM = midregional pro-adrenomedullin; NT-proBNP = N-terminal pro-B-type natriuretic peptide; NYHA = New York Heart Association.

**Figure 4:**
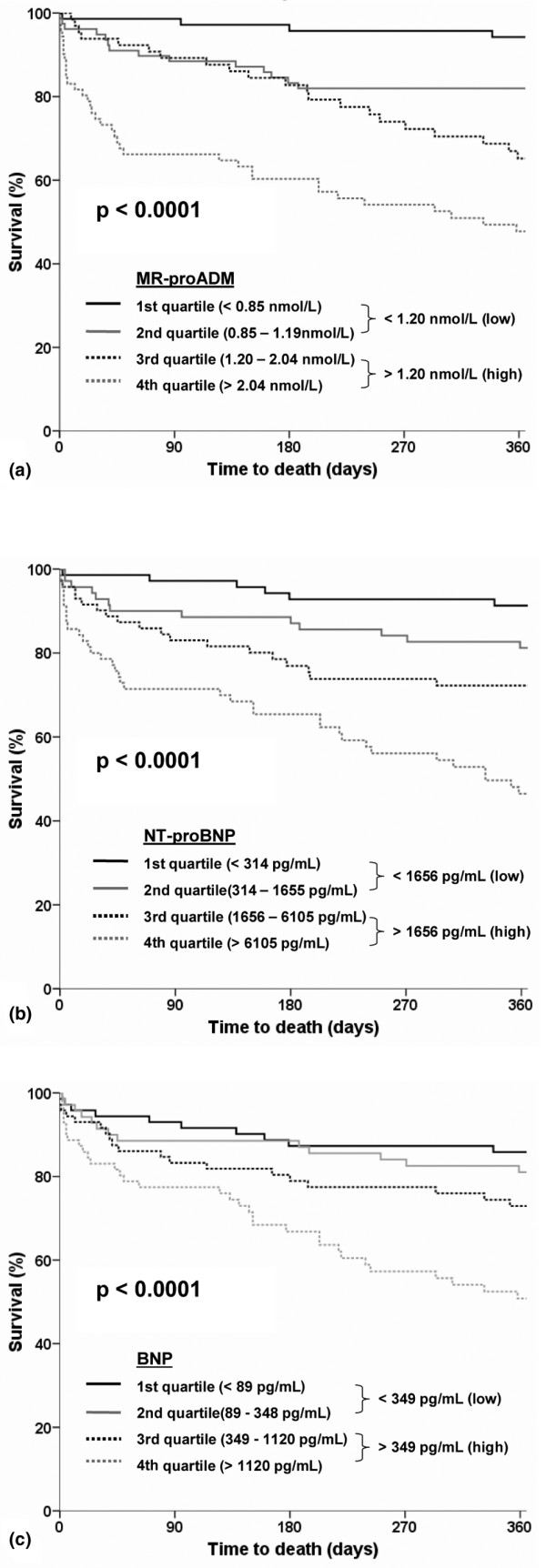
Kaplan-Meier survival curves according to quartiles of **(a)** midregional pro-adrenomedullin, **(b)** N-terminal pro B-type natriuretic peptide and **(c)** B-type natriuretic peptide. BNP = B-type natriuretic peptide; MR-proADM = midregional pro-adrenomedullin; NT-proBNP = N-terminal pro-B-type natriuretic peptide.

Kaplan-Meier curves showed a stepwise increase in one-year all-cause mortality with increasing plasma levels of each of the three biological markers measured at admission: MR-proADM, NT-proBNP and BNP (*P *< 0.001 for all). Thus, one-year all-cause mortality was seemingly different when each of the three biomarkers was above or below the median value (MR-proADM 1.2 nmol/mL; NT-proBNP 1656 pg/mL, and BNP 349 pg/mL; Figure 4).

**Figure 5:**
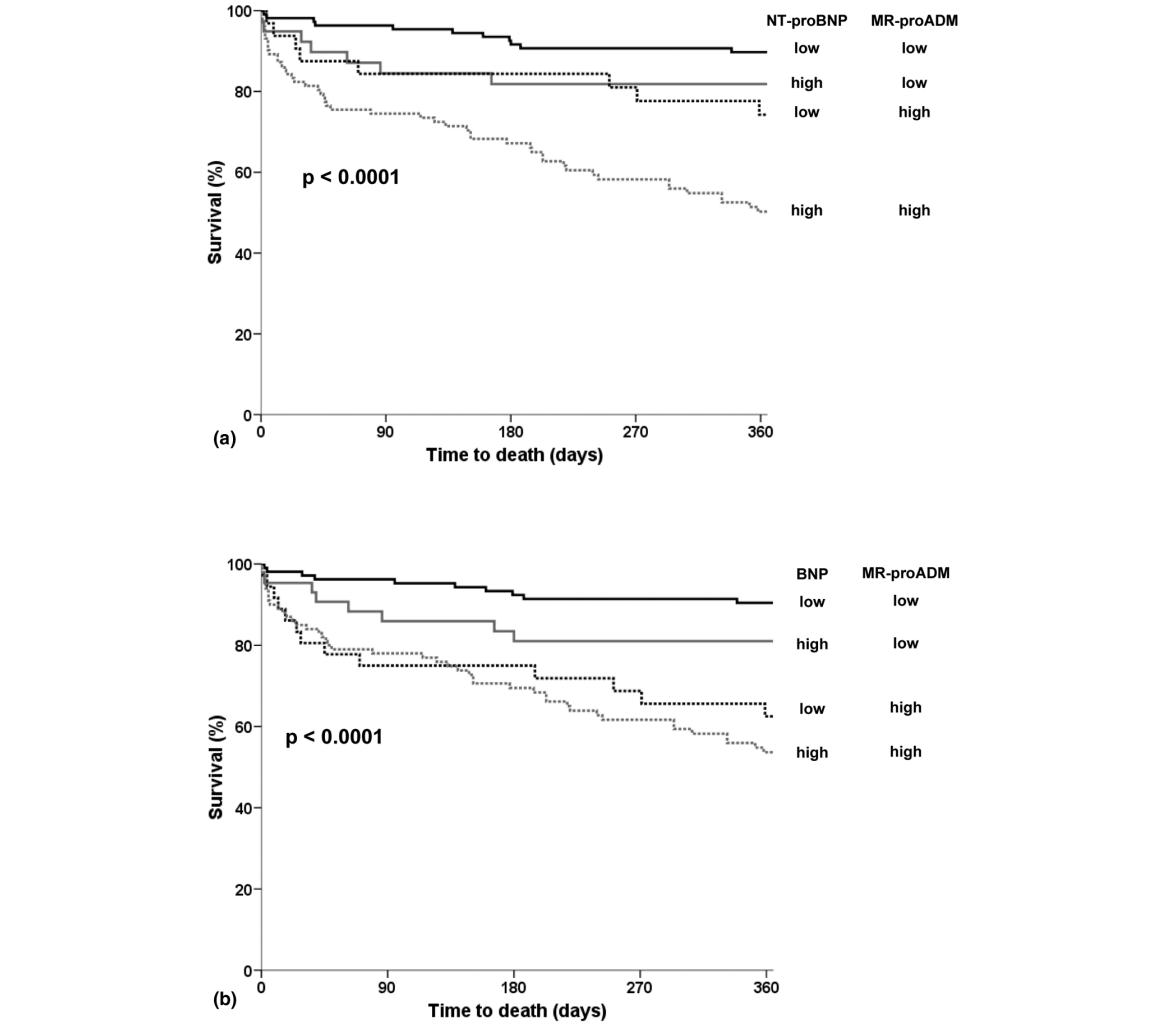
Combined Kaplan-Meier survival curves. **(a) **Combined Kaplan-Meier survival curves according to midregional pro-adrenomedullin (MR-proADM) and N-terminal pro B-type natriuretic peptide (NT-proBNP) values below (low) and above (high) the median. **(b) **Combined Kaplan-Meier survival curves according to MR-proADM and B-type natriuretic peptide (BNP) values below (low) and above (high) the median.

The additional value of combining MR-proADM and NT-proBNP to optimally risk stratify acutely dyspneic patients is shown in Figure 5a. The level of high or low MR-proADM levels (above or below the median) can better stratify patients with either low or high NT-proBNP levels. Accordingly, combined levels of MR-proADM and NT-proBNP did risk stratify acute dyspneic patients into a low (90% one-year survival rate), intermediate (72 to 82% one-year survival rate), or high risk group (52% one-year survival rate; Figure 5a). By contrast, the prognostic value of MR-proADM was only moderate in combination with BNP (Figure 5b).

## Discussion

This study investigated the prognostic potential of MR-proADM in a cohort of unselected patients admitted with acute dyspnea to the ED. The risk stratification of patients with dyspnea admitted to the ED is of paramount importance. An unmet clinical need is to risk stratify this patient population to improve the patient care in the first days of hospitalization. In our study, we found that MR-proADM is a new powerful prognostic marker of death independent of natriuretic peptide levels and regardless of the underlying diagnosis. Furthermore, MR-proADM improved the risk stratification when added to NT-proBNP or to BNP. The combination of MR-proADM and NT-proBNP can best risk stratify acute dyspneic patients into three groups with a low, intermediate, or high-risk of death at one year.

The concept of a biomarker measurement on admission to predict outcome in a variety of diseases has already being studied. Several studies focused on selected patient cohorts with a primary diagnosis of acute coronary syndrome, heart failure, chronic obstructive pulmonary disease, or pulmonary embolism [1,25-27]. Natriuretic peptides have been shown to provide excellent predictive information for patients with acute coronary syndromes, heart failure, and also with sepsis [28-30]. It has been reported that multimarker strategies including natriuretic peptides, troponin, and inflammatory markers are superior to single marker strategies [31,32]. However, little is known about the typical ED population, such as the patient group admitted with acute dyspnea. In clinical practice, the identification of dyspneic patients at highest risk for adverse outcomes remains difficult and largely depends on the underlying cause. Adding to this complexity is the fact that acute dyspnea is often multifactorial and due to cardiac, pulmonary, and inflammatory causes. Specific markers for heart and/or coronary dysfunction may therefore not be ideal to predict the outcome of patients with acute dyspnea in the ED.

Recently, a multimarker strategy (of up to five markers) in patients presenting with acute dyspnea to the ED was suggested [33,34]. These markers included natriuretic peptides, troponin, C-reactive protein, interleukin family member ST2, hemoglobin, and blood urea nitrogen. Both studies have found an increased risk of death in relation to the number of elevated biomarkers.

We hypothesized that MR-proADM could stratify the risk of mortality in patients admitted with acute dyspnea. We found that MR-proADM is a new powerful prognostic marker of death independent of natriuretic peptide levels. Furthermore, we could show that MR-proADM is even superior to BNP in predicting short-term mortality after 30 days. This is in line with studies showing that ADM and the more stable MR-proADM are independent predictors of prognosis in patients with various diseases, such as acute myocardial infarction, heart failure, sepsis, chronic obstructive pulmonary disease, and pneumonia [15-18]. Our study further showed the incremental value of MR-proADM when added to NT-proBNP to risk stratify our acutely dyspneic patients. Indeed, if NT-proBNP was low and MR-proADM was high, the patients had to be classified into the intermediate-risk group instead of the low-risk group. More strikingly, if NT-proBNP was high and MR-proADM was low, patients were classified as intermediate risk instead of high risk. Accordingly, MR-proADM helps in creating an intermediate layer in the risk stratification when combined with NT-proBNP levels in acutely dyspneic patients. However, an additive effect to risk stratify acutely dyspneic patients was only moderate when MR-proADM was combined with BNP.

MR-proADM release in acutely dyspneic patients is most likely related to three possible mechanisms. First, volume overload can activate ADM gene transcription [35] and overexpression of ADM leads to a biologic activity similar to that of natriuretic peptides causing vasodilatation, an increase in cardiac output and induction of natiuresis/diuresis [36]. Second, bacterial endotoxins and proinflammatory cytokines up-regulate ADM gene expression in many tissues [37] in different forms of infection such as pneumonia [15,38]. Third, the kidneys and the lungs play a role in the clearance of ADM. It has been reported that ADM concentrations in aortic blood samples are slightly lower than in pulmonary artery blood samples during selective catheterization [39]. Therefore, impaired removal of circulating ADM in the pulmonary circulation resulting from infection-associated lung injury may partly contribute to the elevation of plasma ADM levels. In contrast, one study suggested that ADM plasma levels in patients with severe lung disease are more likely caused by a systemic production than by a reduced pulmonary clearance [40]. Another possible factor for high ADM plasma levels is endothelial production of ADM triggered by hypoxia [41].

In the present study, the main causes of acute dyspnea were acute heart failure, AECOPD, and pneumonia. Therefore, the above mentioned mechanisms of ADM release reflect the broad spectrum of our acutely dyspneic patients and our findings confirm that the ADM system may be a new powerful candidate for the prediction of adverse outcome in this patient population.

There are several limitations to our study. First, data derived from a single-center study always need to be replicated in larger multi-center studies such as the international, multi-center Biomarkers in ACute Heart failure (BACH) trial. However, our cohort is representative because patient characteristics are comparable with multi-center studies of acute dyspnea [1,42]. Second, we assessed all-cause mortality because classification of death in clinical practice can sometimes be difficult and unreliable [43]. However, exact numbers of all different causes of death could have provided more interesting insights into the pathophysiologic role of the biomarkers.

## Conclusions

In summary, our study suggests that MR-proADM alone or combined with NT-proBNP has the potential to assist clinicians in risk stratifying patients presenting with acute dyspnea regardless of the underlying disease. Indeed, these biomarkers might help emergency physicians to tailor the therapy in view of the relative risk and allocate resources accordingly. Tailored therapy in high-risk patients may include immediate initiation of non-invasive ventilation, consultation of specialists, admission to the intensive care unit, and early and frequent post-discharge visits to prevent relapse and readmission. Whether this risk stratification guided strategy might affect outcome needs to be evaluated prospectively.

## Key messages

• In patients with acute dyspnea, MR-proADM levels are elevated in non-survivors compared with survivors, regardless of the underlying disease.

• MR-proADM on admission predicts 30-day and one-year mortality and seems to be even better than the natriuretic peptides regarding short-term mortality.

• MR-proADM used in addition to natriuretic peptides helps to better risk stratify patients.

## Abbreviations

ADHF: acute decompensated heart failure; ADM: adrenomedullin; AECOPD: acute exacerbation of chronic obstructive pulmonary disease; AUC: area under the curve; BNP: B-type natriuretic peptide; CI: confidence interval; ED: emergency department; MR-proADM: midregional pro-adrenomedullin; NT-proBNP: n-terminal pro-B-type natriuretic peptide; ROC: receivers operating characteristic curves.

## Competing interests

CM has received research support from Abbott, Biosite, Brahms, Roche, and Siemens as well as speaker's honoraria from Abbott, Bayer, Biosite, Brahms, Roche, and Dade Behring. AB is an employee of BRAHMS AG, which is a company developing and marketing *in vitro *diagnostic products, including the MR-proADM assay used in this manuscript. AB also holds patent applications related to this technology, and is a shareholder of BRAHMS AG. NM is an employee of BRAHMS AG. The other co-authors have no competing interests.

## Authors' contributions

MP and CM participated in study concept and design, acquisition of data, analysis and interpretation of data, drafting of the manuscript, critical revision of the manuscript for important intellectual content, had full access to all of the data in the study and take responsibility for the integrity of the data and the accuracy of the data analysis. TB, TR, MN, NS, LB, HU, RB, and MC participated in acquisition of data, analysis and interpretation of data, and critical revision of the manuscript for important intellectual content. NGM, AB, and HF participated in analysis and interpretation of data, and critical revision of the manuscript for important intellectual content. AM participated in analysis, interpretation of data, drafting of the manuscript, and critical revision of the manuscript for important intellectual content. All authors read and approved the final manuscript.
